# A Survey of the Prevalence and Practice Patterns of Human Acellular Nerve Allograft Use

**DOI:** 10.1097/GOX.0000000000001803

**Published:** 2018-08-06

**Authors:** Solomon M. Azouz, Heather D. Lucas, Raman C. Mahabir, Shelley S. Noland

**Affiliations:** From the * Division of Plastic Surgery, Department of Surgery, Mayo Clinic Arizona, Phoenix, Ariz.; † Department of Orthopedic Surgery, Mayo Clinic Arizona, Phoenix, Ariz.

## Abstract

**Background:**

There have been many technical and scientific advances over the last decade in peripheral nerve surgery. Human acellular nerve graft (HANA) has become increasingly popular but current practice patterns among hand surgeons have yet to be defined. Coding practices may not have kept up with this innovation. A 26 question survey of hand surgeons was performed to evaluate the adoption of HANA, and current coding and billing practices. The survey was sent to hand surgeons trained in orthopedic, plastic, general, and neuro surgery. The survey was designed and implemented by the Mayo Clinic Survey Center.

**Results:**

Four hundred sixty-one responses to the survey were received. Most respondents currently use HANA (70%). Of those surgeons who do use HANA, nearly all use it less than 10 times per month (98%). There was no significant difference in the use of HANA across different specialties. There was a significant difference in HANA use depending on practice type with higher use by those in group private practice (57%) compared with academic practice (28%), solo practice (12%), and other practice environment (3%). There was a significant difference in HANA use depending on the number of years in practice. Those in practice less than 5 years used HANA the most (32%), followed by > 20 years in practice (27%), 6–10 years in practice (16%), 16–20 years in practice (14%), and 11–15 years in practice (11%). When asked the Current Procedural Terminology code they would use to bill for the procedure of choice, the most common response was 64910 (nerve repair with synthetic conduit or vein allograft).

**Conclusions:**

HANA has surpassed nerve conduit as the traditional gold standard in our study with nearly 70% of hand surgeons using HANA in their practice and a greater percentage of respondents choosing HANA as their first choice to repair as compared with nerve conduit, nerve autograft, or vein graft. There remains confusion regarding appropriate billing practices for the use of HANA. Due to its common use, a Current Procedural Terminology code should specifically designated for the use of HANA in the hand.

## Abstract 2

Supplemental Digital Content is available in the text.

## INTRODUCTION

Peripheral nerve surgery has enjoyed a renaissance in the last 2 decades.^1^ The use of human acellular nerve allograft (HANA), the popularization of nerve transfers, and modern postoperative rehabilitation have improved outcomes for patients with devastating nerve injuries.^1^ HANA was approved by Food and Drug Administration (FDA) in 2007, and over the last decade its popularity has increased, particularly for short sensory nerve gaps.^2–7^ Advantages include ease of use, lack of donor-site morbidity, and decreased operative time.^2–7^ Although there are numerous studies demonstrating clinical outcomes of HANA,^2–7^ there have been no published studies of its use by hand surgeons. Furthermore, coding remains inconsistent as there is currently no specific Current Procedural Terminology (CPT) code for HANA use. A survey was designed to identify the prevalence and practice patterns of HANA use among hand surgeons.

**Table:**
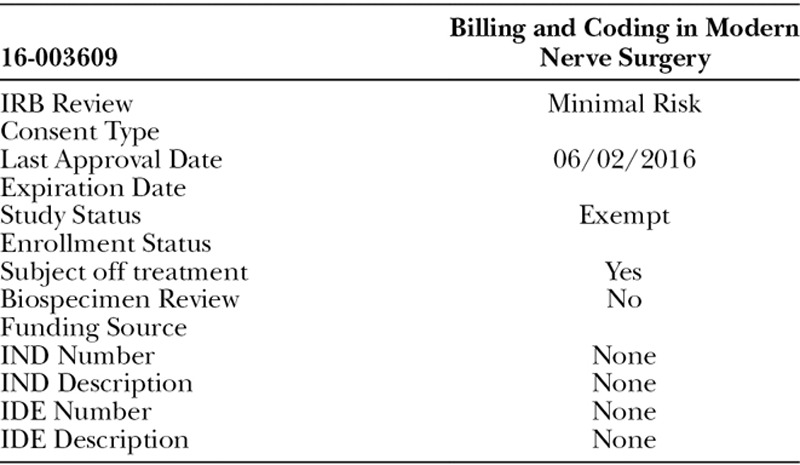


## MATERIALS AND METHODS

After institutional review board approval, a 26 question survey was designed and distributed to all members of the American Society for Surgery of the Hand and the American Association of Hand Surgery. These 2 societies represent the largest contingency of hand surgeons in the United States. The study was determined to be exempt by the Mayo Clinic Institutional Review Board 16-003609, and respondents consented to participate via an e-mailed written consent prompt. The survey was created and distributed electronically through the Mayo Clinic Survey Research Center using Qualtrics Survey Software. The survey questions included demographics, peripheral nerve surgery practice, HANA use, and a specific clinical scenario targeting the management of a digital nerve gap (**see appendix, Supplemental Digital Content 1**, which shows the entire survey instrument used in the collection of data from hand surgeons, http://links.lww.com/PRSGO/A790). Data were collected, and statistical analysis performed via the Mayo Clinic Survey Research Center. Responses for each field were tallied and a multivariate logistic regression model was used to identify factors associated with HANA use. All statistical analysis was performed using SAS version 9.4 software (SAS Institute Inc., Cary, N.C.), and values of *P* < 0.05 were considered statistically significant.

## RESULTS

### Demographics

**Fig. 1.**
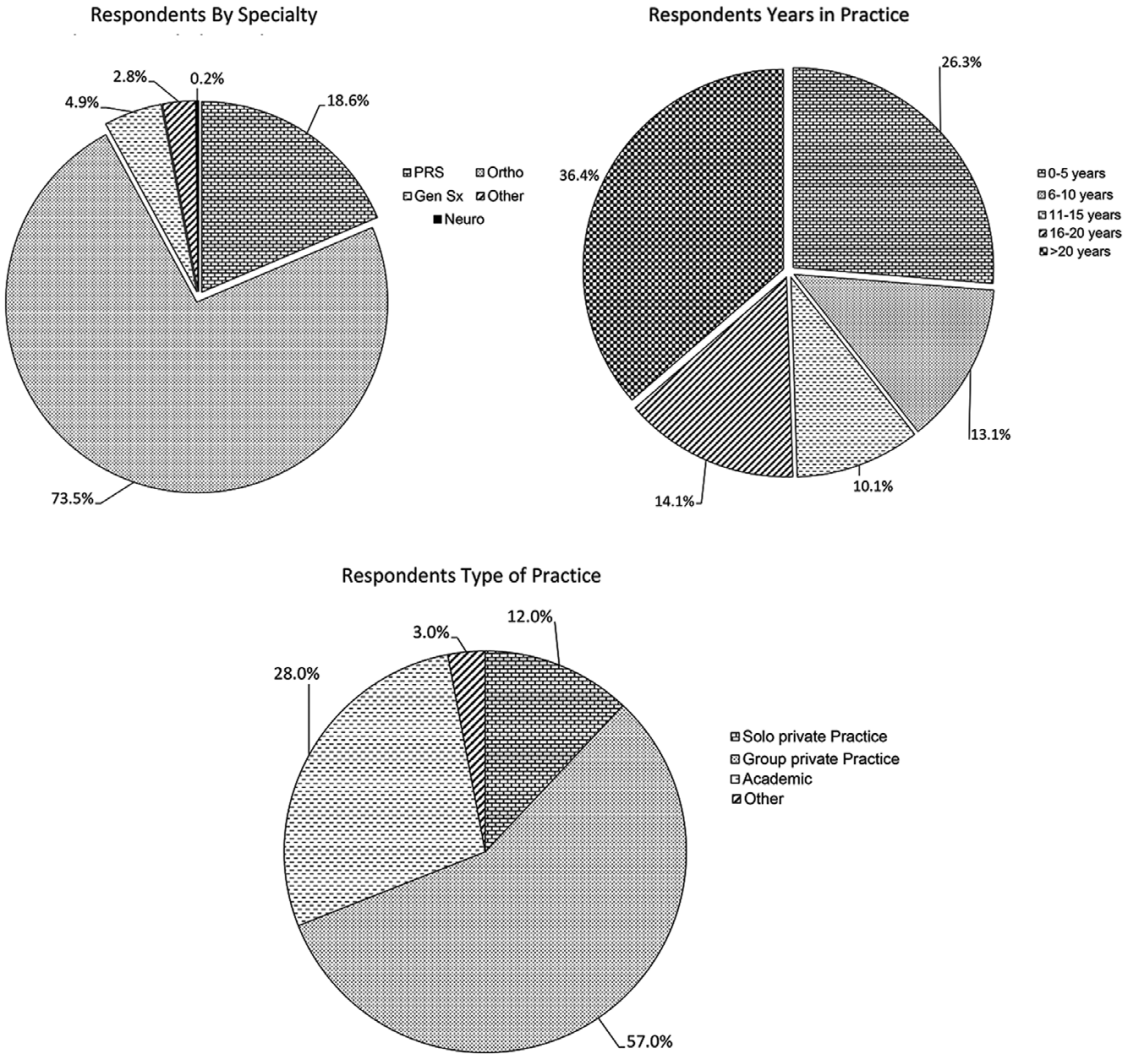
Respondents demographics. Professional characteristics of the survey population. A, Distribution of respondents by specialty. B, Distribution of years in practice of surgeons. C, Distribution of respondents by type of practice. (a) Respondents By Specialty; (b) Respondents Years in Practice; (c) Respondents Type of Practice.

Four hundred sixty-one responses to the survey were received of the 4,045 American Society for Surgery of the Hand and 1,131 American Association of Hand Surgery members. It was not possible to discern which survey recipients had dual memberships and therefore received the survey twice. The majority of respondents were trained in orthopedic surgery (76%) or plastic surgery (19%), followed by general surgery (5%) and neurosurgery (< 1%; Fig. 1A).

Of the respondents, 39% were in practice less than 10 years, 25% 11–20 years and 36% greater than 20 years (Fig. 1B). Most respondents were associated with a group private practice (57%), followed by full-time faculty at an academic institution (28%), solo practice (12%), or other practice environment (3%; Fig. 1C). For those surgeons who completed a fellowship, 98% completed a hand surgery fellowship, 11% completed a peripheral nerve fellowship, and 7% completed a different fellowship (ie, microsurgery; Table 1). Some respondents completed multiple fellowships.

**Table 1.**
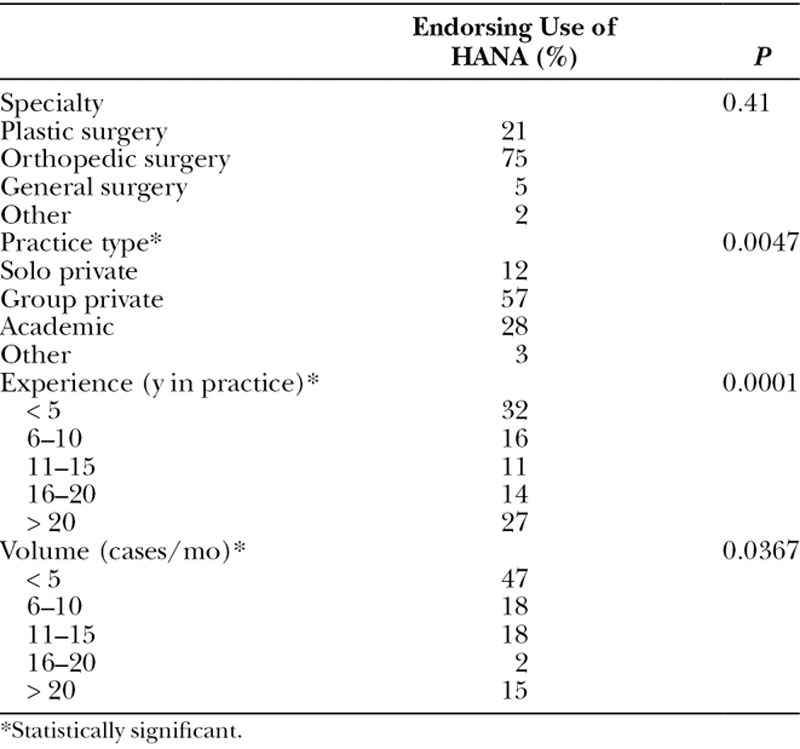
Prevalence of HANA Use Among Hand Surgeons

### Peripheral Nerve Surgery Practice and HANA Use

When asked about the frequency of peripheral nerve surgeries performed, most perform fewer than 5 surgeries per month (47%; Table 1). Most respondents currently use HANA (70%). Of those surgeons who do use HANA, nearly all use it less than 10 times per month (98%). There was no significant difference in the use of HANA across different specialties (*P* = 0.41; Table 1). There was a significant difference in HANA use depending on practice type with higher use by those in group private practice (57%) compared with academic practice (28%), solo practice (12%), and other practice environment (3%; *P* = 0.0047). There was a significant difference in HANA use depending on the number of years in practice (*P* = 0.0001). Those in practice less than 5 years used HANA the most (32%), followed by > 20 years in practice (27%), 6–10 years in practice (16%), 16–20 years in practice (14%), and 11–15 years in practice (11%). There was a significant difference in HANA use depending on the number of peripheral nerve cases performed per month (*P* = 0.0367) with those performing less than 5 peripheral nerve cases per month having the highest percentage use (37%), followed by 6–10 cases (23%), 11–15 cases and > 20 cases (both 20%), and 16–20 cases (0%). Figure 1 shows the use of HANA depending on specialty, number of years in practice, number of nerve, and type of practice.

### Clinical Scenario

A 35-year-old male presents with a dog bite to the right middle finger resulting in a traumatic laceration of the radial digital nerve at the level of proximal interphalangeal joint. After exploration and trimming here is a 2-cm gap of the nerve.

**Fig. 2.**
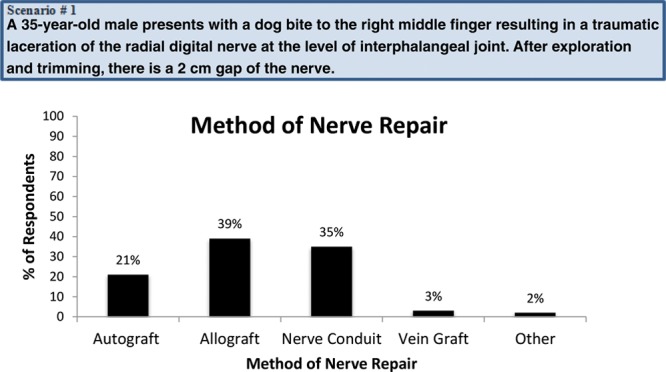
Method of nerve repair selected for a 2-cm digital nerve gap.

When presented with the clinical scenario above, 39% of respondents would use HANA, 35% nerve conduit, 21% nerve autograft, 3% vein graft as conduit, and 2% would utilize some other method (Fig. 2). When asked the CPT code they would use to bill for their procedure of choice, the most common response was 64910 (nerve repair with synthetic conduit or vein allograft, 49%), followed by 64890 (27%), 64831 (19%), 64999 (7%), 64834 and 64911 (each 1%). Table 2 demonstrates the frequency of CPT codes selected and CPT code selected by preferred nerve repair technique.

**Table 2.**
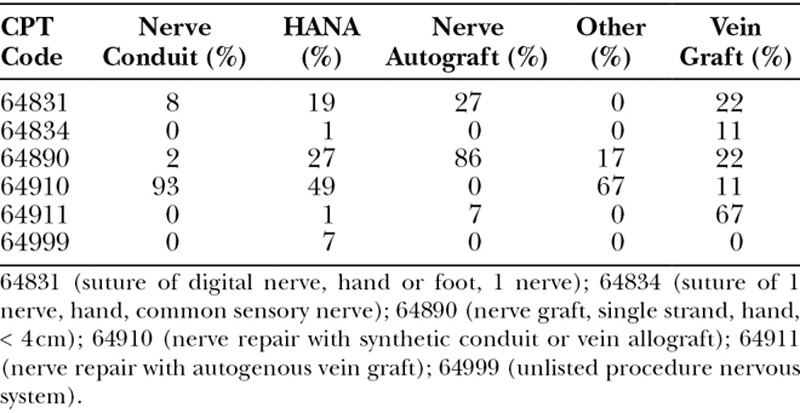
CPT Code by Procedure Chosen

**Table 3.**
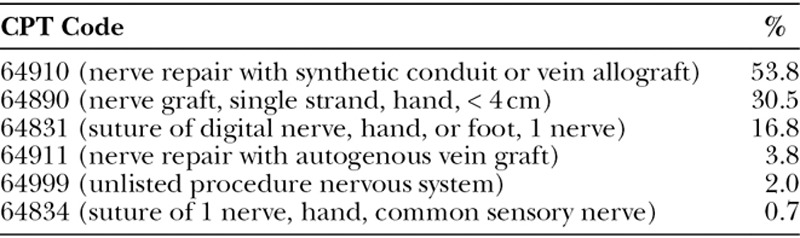
Frequency of CPT Codes Chosen by Respondents

## DISCUSSION

Nerve repair may date as far back as to the time of Hippocrates.^8^ The use of the operating microscope, knowledge of the internal topography of nerves, and clinical and basic science research have advanced the management of nerve injury, recovery, and repair.^9^ HANA is an important advancement in nerve surgery and offers an additional tool in the armamentarium of a hand surgeon. HANA was approved by the FDA in 2007. Since then, its use has increased in frequency and more data continue to emerge.^2–7,10–12^ HANA theoretically combines the off-the-shelf access of conduits with the structural axons of autograft. Advances in tissue processing in the last decade have overcome the immunogenicity associated with prior allografts, permitting regular use.^4^ Systematic reviews have demonstrated the success of HANA in reconstructing short gaps.^4,13^ Some recent studies suggest that larger gaps may also be reconstructed with HANA.^6,14,15^

Over 450 hand surgeons responded to our survey and were of similar distribution to national demographics and nearly 70% use HANA in their hand surgery practice. Nearly all use HANA less than 10 times per month. The use of HANA is commonplace in contemporary hand surgery practice, regardless of specialty, number of years in practice, type of practice, or fellowship training.

Although we had an even distribution of responses across experience levels, there was not an even distribution of HANA use. There was a bivariate distribution depending on the number of years in practice, with about 32% of those in practice less than 5 years using HANA and 27% of those in practice more than 20 years using HANA. The number of respondents in each bivariate group was similar. We anticipated that surgeons in practice less than 5 years would be more likely to use HANA because they would have completed at least a portion of their residency and fellowship training after FDA approval of HANA and were likely exposed to HANA during that time. We did not anticipate the increased use of HANA in those in practice > 20 years, and we do not have an explanation for this. Each of these bivariate groups (practice < 5 years versus > 20 years) uses HANA at nearly double the rate of those in practice 6–20 years. Academic practice surgeons (28%) may have lower usage of allograft because there are residents and fellows to harvest the nerve and close the donor site. Solo practitioners (12%) may use HANA most infrequently because those surgeons might work in a surgery center where allograft is not available, whereas those working in a group practice (57%) likely have a presence in a large hospital where allograft is stocked and readily available.

When presented with a case scenario of a 2-mm digital nerve gap, with the highest percentage of respondents choosing HANA (39%) as their repair method of choice, followed by nerve conduit (35%) and nerve autograft (21%). In this study, HANA surpassed nerve conduit traditional gold standard,^1,16^ nerve autograft in the scenario, but this is limited by a low response rate and variability of choice in different professional settings. There are 2 clinical studies sponsored by AxoGen, Inc., (Alachua, Fla.) and a literature review that suggests that HANA may be a better and more reliable option than nerve conduit.^6,17,18^ It is surmised that this is related to the inherent nerve structure within HANA when compared with the empty chamber of nerve conduits.

This study only looked at HANA use in a short 2-cm sensory nerve gap. The published data confirm that this is an appropriate use of HANA. Although there are some case studies in the literature,^19,20^ larger studies are needed to confirm the appropriateness of HANA use in mixed nerves or in nerve gaps longer than 3 cm.^21–25^

There has been some confusion regarding appropriate coding practice for the use of HANA. About half of respondents (49%) who stated that they would repair the nerve with HANA would use CPT code 64910 (nerve repair with synthetic conduit or vein allograft). Twenty-seven percentage would use CPT code 64890 (nerve graft, single strand, hand, < 4 cm). Because there is no CPT code specifically designated for the use of HANA, these are the alternatives that hand surgeons have identified. As demonstrated in Table 2, there is variability in coding for the various nerve repair techniques. Respondents who selected nerve conduit or nerve autograft had relative consistency in their coding choices, whereas those who selected HANA or vein graft did not. It would be useful to have a CPT code specifically designated for the use of HANA.

The limitations of this study include the low response rate and the singularity and simplicity of the clinical scenario. We did not look at other types of nerve repair including mixed nerves and longer nerve gaps. However, this is the first published article to shed light on the prevalence and practice patterns of HANA use among hand surgeons.

## CONCLUSIONS

Over one-third of surgeons now report using HANA in their practice, and in the repair of a short sensory nerve gap, HANA was the repair of choice in respondents. There remains a large variability in the CPT coding for HANA.

## Supplementary Material
