# Supplementary material for: A Survey of the Prevalence and Practice Patterns of Human Acellular Nerve Allograft Use
Source: Plast Reconstr Surg Glob Open. 2018 Aug 6;6(8):e1803. doi: 10.1097/GOX.0000000000001803 (PMC6143321; doi:10.1097/GOX.0000000000001803)
Supplement: Supplementary file 1 [file gox-6-e1803-s001.pdf]

Appendix Supplementary

HANA Prevalence and Practice Patterns Survey

1. Specialty (Check all that apply)

a) Plastic Surgery

b) Neurosurgery

c) Orthopedic Surgery

d) General Surgery

e) Other______________

2. Years Practicing in Specialty

a) 0 - 5

b) 6 - 10

c) 11 - 15

d) 16 – 20

e) > 20

3. Primary Type of Practice (Check one)

a) Solo Private Practice

b) Solo Private Practice with Academic Affiliation

c) Group Private Practice

d) Group Private Practice with Academic Affiliation

e) HMO

f) HMO with Academic Affiliation

g) Full-Time Faculty at Academic Institution

h) Other _______________

4. Fellowship (Check all that apply)

a) Hand Surgery

b) Peripheral Nerve Surgery

c) Other _______________

5. How many nerve surgeries do you perform per month?

a) < 5

b) 6 – 10

c) 11 – 15

d) 16 – 20

e) > 20

6. Do you use HANA?

a) Yes

b) No

7. If you do use HANA, how many times per month do you use it?

a) < 5

b) 6 -10

c) 11 – 15

d) 16 – 20

e) > 20

8. Do you perform nerve transfers?

a) Yes

b) No

9. If you do perform nerve transfers, how many times a month do you perform them?

a) < 5

b) 6 – 10

c) 11 – 15

d) 16 – 20

e) > 20

Scenario # 1

A 35 year male presents with a dog bite to the right middle finger resulting in a traumatic laceration of the radial digital nerve at level of proximal interphalangeal joint. After exploration and trimming there is a 2 cm gap of the nerve.

10. How do you repair the nerve?

a) Nerve Autograft

b) HANA

c) Commercially Available Nerve Conduit

d) Vein Graft as Conduit

e) Other

21. How do you bill for the nerve repair? Please select all the CPT code(s) you would use to describe the procedure performed

for the patient in Scenario 1.

a) 64831 (Suture of digital nerve, hand or foot; 1 nerve)

b) 64834 (Suture of 1 nerve; hand or foot, common sensory nerve)

c) 64890 (Nerve graft – includes obtaining graft – single strand, hand or foot; up to 4 cm length

d) 64910 (Nerve repair; with synthetic conduit or vein allograft - eg, nerve tube – each nerve)

e) 64911 (Nerve repair; with autogenous vein graft - includes harvest of vein graft – each nerve)

f) 64999 (Unlisted procedure, nervous system)

g) +69990 (ADD ON CODE MUST USE IN ADDITION TO ANOTHER PRIMARY CODE: Use of operating microscope)

h) Other

How much of your own time is required per patient treated for each of the following steps in patient care related to the CPT code(s) for Scenario 1?

<u>Day of procedure</u>

12. Pre-service time related to primary procedure (Includes any services provided by the physician the day before the procedure up to and until the time of the procedure. It may include review of records, communications with other professionals, and obtaining consent for the procedure. It also includes the time to dress, scrub and wait before the procedure, and any time spent positioning the patient or equipment for the procedure.)

a) 0-10 min

b) 11-20 min

c) 21-30 min

d) 31-40 min

e) 41-50 min

f) 51-60 min

13. For the referenced service (codes) in Scenario 1 rate the average pre service complexity on a scale of 1 to 5 (1=low; 3=medium; 5=high).

Pre-service complexity

a) 1

b) 2

c) 3

d) 4

e) 5

14.. Intra – service time (skin to skin):

a) 0-30 min

b) 31-60 min

c) 61-90 min

d) 91-120 min

e) 121-150 min

f) 151-180 min

15. For the referenced service (codes) in Scenario 1 rate the average intra service complexity on a scale of 1 to 5 (1=low; 3=medium; 5=high).

Intra-service complexity:

a) 1

b) 2

c) 3

d) 4

e) 5

16. Post-service time related to primary procedure. (Includes non "skin-to-skin" work in the OR, patient stabilization in the recovery room, and communicating with the patient and other professionals including written and telephone reports and orders.)

a) 0-10 min

b) 11-20 min

c) 21-30 min

d) 31-40 min

e) 41-50 min

f) 51-60 min

17. For the referenced service (codes) in Scenario 1 rate the average post service complexity on a scale of 1 to 5 (1=low; 3=medium; 5=high).

Post-service complexity:

a) 1

b) 2

c) 3

d) 4

e) 5

For Scenario 1 rate the intensity for each component listed on a scale of 1 to 5. (1=low; 3=medium; 5=high) Mental Effort and Judgment

18. The number of possible diagnoses and/or the number of management options that must be considered

a) 1

b) 2

c) 3

d) 4

e) 5

19. The amount and/or complexity of medical records, diagnostic tests, and/or other information that must be obtained reviewed and analyzed

a) 1

b) 2

c) 3

d) 4

e) 5

20. Urgency of medical decision making

a) 1

b) 2

c) 3

d) 4

e) 5

Technical Skill/Physical Effort

21. Technical skill required

a) 1

b) 2

c) 3

d) 4

e) 5

22. Physical effort required

a) 1

b) 2

c) 3

d) 4

e) 5

Psychological Stress

23. The risk of significant complications, morbidity and /or mortality

a) 1

b) 2

c) 3

d) 4

e) 5

24. How much the outcome depends on skill and judgment of physician

a) 1

b) 2

c) 3

d) 4

e) 5

25. The estimated risk of malpractice suit with poor outcome

a) 1

b) 2

c) 3

d) 4

e) 5

26. How many work relative value units (wRVUs) do you think the procedure in Scenario 1 is worth? (Refer to RVU Code Table 1)

1) 0-5

2) 6-10

3) 11-15

4) 16-20

5) 21-25

6) 26-30

7) 31-35

8) 36-40

RVU Code Table 1	
Code	RVU
64831	9.16
64834	10.81
64856	15.07
64890	16.24
64910	11.39
64911	14.39
